# Treatment With Avacopan in ANCA–Associated Vasculitis With Kidney Involvement

**DOI:** 10.1016/j.ekir.2025.05.041

**Published:** 2025-06-02

**Authors:** Duvuru Geetha, Frank B. Cortazar, Annette Bruchfeld, Andreas Kronbichler, Alexandre Karras, Georges N. Nakhoul, Peter A. Merkel, Sarah Bray, Alana M. Bozeman, David R.W. Jayne, ADVOCATE Study Group, C. Au Peh, A. Chakera, B. Cooper, J. Kurtkoti, D. Langguth, V. Levidiotis, G. Luxton, P. Mount, D. Mudge, E. Noble, R. Phoon, D. Ranganathan, A. Ritchie, J. Ryan, M. Suranyi, A. Kronbichler, A. Rosenkranz, K. Lhotta, N. Demoulin, C. Bovy, R. Hellemans, J. Hougardy, B. Sprangers, K. Wissing, C. Pagnoux, S. Barbour, S. Brachemi, S. Cournoyer, L. Girard, L. Laurin, P. Liang, D. Philibert, M. Walsh, C. Rigothier, J. Augusto, A. Belot, D. Chauveau, D. Cornec, N. Jourde-Chiche, M. Ficheux, P. Zaoui. Paris, A. Karras, A. Klein, F. Maurier, R. Mesbah, O. Moranne, A. Neel, T. Quemeneur, D. Saadoun, B. Terrier, P. Zaoui, M. Schaier, U. Benck, R. Bergner, M. Busch, J. Floege, F. Grundmann, H. Haller, M. Haubitz, B. Hellmich, J. Henes, B. Hohenstein, C. Hugo, C. Iking-Konert, F. Arndt, T. Kubacki, I. Kotter, P. Lamprecht, T. Lindner, J. Halbritter, H. Mehling, U. Schönermarck, N. Venhoff, V. Vielhauer, O. Witzke, I. Szombati, G. Szucs, G. Garibotto, F. Alberici, E. Brunetta, L. Dagna, S. De Vita, G. Emmi, A. Gabrielli, L. Manenti, F. Pieruzzi, D. Roccatello, C. Salvarani, H. Dobashi, T. Atsumi, S. Fujimoto, N. Hagino, A. Ihata, S. Kaname, Y. Kaneko, A. Katagiri, M. Katayama, Y. Kirino, K. Kitagawa, A. Komatsuda, H. Kono, T. Kurasawa, R. Matsumura, T. Mimura, A. Morinobu, Y. Murakawa, T. Naniwa, T. Nanki, N. Ogawa, H. Oshima, K. Sada, E. Sugiyama, T. Takeuchi, H. Taki, N. Tamura, T. Tsukamoto, K. Yamagata, M. Yamamura, P. van Daele, J. de Zoysa, A. Rutgers, Y. Teng, R. Walker, I. Chua, M. Collins, K. Rabindranath, J. de Zoysa, M. Svensson, B. Grevbo, S. Kalstad, M. Little, M. Clarkson, E. Molloy, I. Agraz Pamplona, J. Anton, V. Barrio Lucia, S. Ciggaran, M. Cinta Cid, M. Diaz Encarnacion, X. Fulladosa Oliveras, M. Jose Soler, H. Marco Rusinol, M. Praga, L. Quintana Porras, A. Segarra, A. Bruchfeld, M. Segelmark, I. Soveri, E. Thomaidi, K. Westman, T. Neumann, M. Burnier, T. Daikeler, J. Dudler, T. Hauser, H. Seeger, B. Vogt, D. Jayne, J. Burton, R. Al Jayyousi, T. Amin, J. Andrews, L. Baines, P. Brogan, B. Dasgupta, T. Doulton, O. Flossmann, S. Griffin, J. Harper, L. Harper, D. Kidder, R. Klocke, P. Lanyon, R. Luqmani, J. McLaren, D. Makanjuola, L. McCann, A. Nandagudi, S. Selvan, E. O'Riordan, M. Patel, R. Patel, C. Pusey, R. Rajakariar, J. Robson, M. Robson, A. Salama, L. Smyth, J. Sznajd, J. Taylor, P. Merkel, A. Sreih, E. Belilos, A. Bomback, J. Carlin, Y. Chang Chen Lin, V. Derebail, S. Dragoi, A. Dua, L. Forbess, D. Geetha, P. Gipson, R. Gohh, G.T. Greenwood, S. Hugenberg, R. Jimenez, M. Kaskas, T. Kermani, A. Kivitz, C. Koening, C. Langford, G. Marder, A. Mohamed, P. Monach, N. Neyra, G. Niemer, J. Niles, R. Obi, C. Owens, D. Parks, A. Podoll, B. Rovin, R. Sam, W. Shergy, A. Silva, U. Specks, R. Spiera, J. Springer, C. Striebich, A. Swarup, S. Thakar, A. Tiliakos, Y. Tsai, D. Waguespack, M. Chester Wasko

**Affiliations:** 1 Department of Medicine, Johns Hopkins University School of Medicine, Baltimore, Maryland, USA; 2 New York Nephrology Vasculitis and Glomerular Center, Albany, New York, USA; 3 Department of Health, Medicine and Caring Sciences, Linköping University, Linköping, Sweden; 4 Department of Renal Medicine, Karolinska University Hospital and CLINTEC Karolinska Institutet, Stockholm, Sweden; 5 Department of Internal Medicine IV, Nephrology and Hypertension, Medical University Innsbruck, Innsbruck, Austria; 6 Department of Medicine, University of Cambridge, Cambridge, UK; 7 Université Paris Cité, Paris, France; 8 Department of Nephrology, Hôpital Européen Georges Pompidou, APHP, Paris, France; 9 Department of Kidney Medicine, Cleveland Clinic, Cleveland, Ohio, USA; 10 Department of Medicine, Perelman School of Medicine, University of Pennsylvania, Philadelphia, Pennsylvania, USA; 11 Department of Biostatistics, Epidemiology, and Informatics, Perelman School of Medicine, University of Pennsylvania, Philadelphia, Pennsylvania, USA; 12 Department of Biostatistics, Amgen Ltd, Cambridge, UK; 13 Department of Medical Affairs, Amgen Inc., Thousand Oaks, California, USA

**Keywords:** ANCA, complement, granulomatosis with polyangiitis (GPA), hematuria, microscopic polyangiitis (MPA), proteinuria

## Abstract

**Introduction:**

Kidney disease impacts long-term outcomes of patients with granulomatosis with polyangiitis (GPA) and microscopic polyangiitis (MPA). This *post hoc* analysis evaluated the effect of avacopan in a subgroup of patients with GPA or MPA and kidney involvement at baseline from the ADVOCATE trial.

**Methods:**

The analysis included a study population of 268 patients (of 330 patients, 81.2%). Key efficacy outcomes were remission at week 26, sustained remission at week 52, and relapse after remission through week 52. Changes in estimated glomerular filtration rate (eGFR) were analyzed overall and stratified by baseline eGFR categories (≥ 90, 60–89, 45–59, 30–44, and 15–29 ml/min per 1.73 m^2^). Additional outcomes were changes in albuminuria and hematuria, glucocorticoid (GC) use, glucocorticoid toxicity index (GTI), and safety.

**Results:**

Remission at week 26 and sustained remission at week 52, respectively, were respectively achieved by 99 of 134 (73.9%) and 91 of 134 (67.9%) patients in the avacopan group and 95 of 134 (70.9%) and 76 of 134 (56.7%) in the prednisone taper group. Relapse rate after remission was lower in the avacopan than in the prednisone taper group (9.4% vs. 20.9%; hazard ratio [95% confidence interval, CI]: 0.43 [0.22–0.85]). Recovery of kidney function, speed of reduction in albuminuria and hematuria, and changes in GTI favored the avacopan group. No new safety issues were reported for this subset of patients.

**Conclusion:**

In patients with GPA or MPA with kidney involvement, treatment with an avacopan regimen compared with a prednisone taper regimen achieved similar rates of remission, improved recovery of kidney function, led to faster reduction in albuminuria and hematuria, and lowered GC-related toxicity, with an acceptable safety profile.

## Graphical abstract

**Figure:**
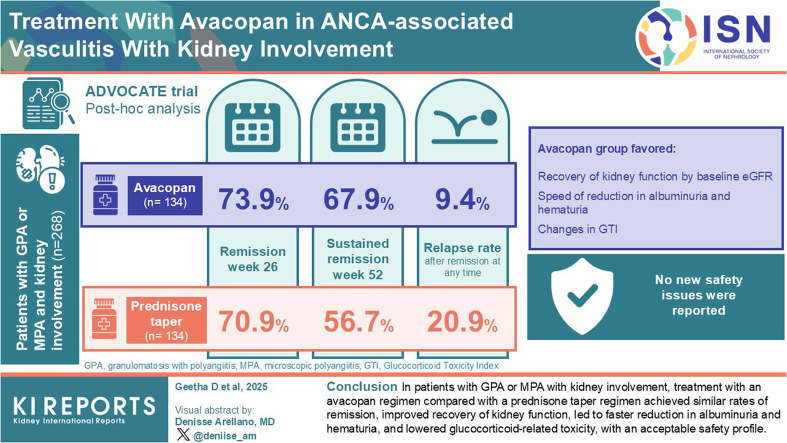

GPA and MPA are subtypes of antineutrophil cytoplasmic antibody (ANCA)-associated vasculitis that frequently manifest with kidney involvement.^1^, ^2^, ^3^, ^4^, ^5^, ^6^, ^7^ In general, patients with GPA or MPA often experience deterioration in health-related quality of life (HRQoL) and mortality because of organ damage,^8^, ^9^, ^10^ as well as toxicity from medications used to treat the disease, including from the long-term use of GCs.^11^, ^12^, ^13^

Avacopan is an orally administered small-molecule complement 5a receptor-1 antagonist that inhibits the interaction of complement 5a with complement 5a receptor-1, blocking neutrophil chemoattraction and activation.^14^ In the phase 3 ADVOCATE randomized trial in patients with GPA or MPA who received background immunosuppressive induction therapy with cyclophosphamide (followed by therapy to maintain remission with azathioprine or mycophenolate mofetil) or rituximab, avacopan was noninferior to a prednisone taper in achieving remission at week 26 and superior with regard to sustained remission at week 52.^14^ Because the severity of kidney disease is a key factor in the long-term outcomes of patients with GPA or MPA, this *post hoc* analysis of the ADVOCATE trial was conducted to describe results in the subgroup of patients with GPA or MPA and kidney involvement at baseline.

## Methods

### Study Design and Patients

Patients enrolled in ADVOCATE were included in this analysis if they had kidney involvement of GPA or MPA, defined as presence of baseline kidney manifestations according to the Birmingham Vasculitis Activity Score (BVAS) version 3 renal domains^15^ as assessed by the investigator. ADVOCATE was a multicenter, randomized, double-blind, double-dummy, active-controlled trial (NCT02994927) that compared avacopan with a prednisone taper in combination with additional immunosuppressive medications in patients with newly diagnosed or relapsed GPA or MPA.^14^ Details of the ADVOCATE trial have been previously published,^14^ and are summarized in the Supplementary Methods.

ADVOCATE was conducted in compliance with the Declaration of Helsinki, the International Council for Harmonization Guidelines for Good Clinical Practice, and local country regulations. The study protocols were approved by each center’s institutional review board or independent ethics committee. Patients or their parent or guardian provided written informed consent before study initiation.

### Efficacy Outcomes

This article reports an exploratory *post hoc* analysis of overall and kidney-specific outcomes in patients with GPA or MPA and kidney involvement at baseline. The overall outcomes were remission at week 26 (defined as a BVAS of 0 and no receipt of GCs for the treatment of GPA or MPA 4 weeks before week 26) and sustained remission (defined as remission at week 26 and a BVAS of 0 at week 52 with no receipt of GCs for the treatment of GPA or MPA 4 weeks before week 52 and no relapse between weeks 26 and 52). Relapse was defined as a return of active vasculitis after the previous achievement of a BVAS of 0 at any time that involved 1 or 2 minor BVAS items for at least 2 consecutive study visits, at least 1 major BVAS item, or at least 3 minor BVAS items. Additional exploratory analyses included the proportion of patients experiencing a first relapse after achieving BVAS of 0, a kidney relapse (defined as a recurrence of a kidney manifestation of vasculitis [per the BVAS] after kidney manifestations of vasculitis had resolved, irrespective of any clinical intervention), and a kidney relapse after remission (defined as recurrence of a kidney manifestation of vasculitis [per the BVAS] at weeks 39 and/or 52 after the primary endpoint of remission was achieved at week 26).

eGFR (in ml/min per 1.73 m^2^) was calculated using the serum creatinine-based Modification of Diet in Renal Disease formula for adults,^16^ the Japanese equation for Japanese adults,^17^ and the modified Schwartz equation for adolescents.^18^ eGFR was analyzed as measured irrespective of whether dialysis was initiated on study. Change in eGFR was analyzed overall and stratified by baseline eGFR categories of ≥ 90 ml/min per 1.73 m^2^, 60 to 89 ml/min per 1.73 m^2^, 45 to 59 ml/min per 1.73 m^2^, 30 to 44 ml/min per 1.73 m^2^, 15 to 29 ml/min per 1.73 m^2^, and < 15 ml/min per 1.73 m^2^ (eligibility deviation), wherever applicable.^19^ An additional analysis evaluated eGFR change as shifts in eGFR category over the 52 weeks of the trial. For albuminuria, the mean urinary albumin-to-creatinine ratio (UACR) and the percentage of patients with UACR > 300 mg/g creatinine^20^ were determined, different from earlier publications that determined UACR > 10 mg/g creatinine. Hematuria was categorized by number of red blood cells (RBCs)/high powered field (hpf) on central laboratory–assessed urinalysis as none, 1 to < 10, 10 to 29, 30 to 49, 50 to 75, and > 75 RBCs/hpf.

Further exploratory analyses included use of GCs (presented as mg prednisone equivalent), and GTI.^21^ Lower scores indicate lesser severity of toxic effects both in the GTI cumulative worsening score and the GTI aggregate improvement score. Change from baseline in HRQoL was assessed using the 36-Item Short Form Health Survey (SF-36) questionnaire version 2.^22^

### Safety Outcomes

Safety outcomes included incidence of adverse events (AEs) and serious AEs. Data were collected and coded using the Medical Dictionary for Regulatory Activities version 19.1^23^ and graded according to the Common Terminology Criteria for Adverse Events version 5.0.^24^

### Statistical Analysis

Exploratory data were summarized descriptively and stratified by treatment group. All analyses were conducted in the modified intention-to-treat population, defined as all randomly assigned patients who received at least 1 dose of trial medication. The proportions of patients achieving disease remission at week 26 and sustaining disease remission at week 52 were determined. CIs for proportions were calculated using the Clopper-Pearson method. CIs for difference in proportions were calculated using the Wald method. Missing data were imputed as not achieving remission (week 26) or sustained remission (week 52). The Cox proportional hazard model was used to estimate the hazard ratio of time to relapse and time to kidney relapse. Proportional hazards were verified by incorporating a time-varying covariate in the Cox regression model by creating an interaction of the treatment groups and log of the time to relapse. For changes from baseline, least squares (LS) mean and SEM were calculated from mixed-effects models for repeated measures with treatment group, visit, and treatment-by-visit interaction as factors and baseline as a covariate. Longitudinal measurements for the same patients were considered as repeated measure units. Remission and change from baseline were stratified by baseline eGFR category (> 90, 60–89, 45–59, 30–44, and 15–29 ml/min per 1.73 m^2^). GTI aggregate improvement score, GTI cumulative worsening score, and HRQoL outcomes were analyzed using mixed-effects models for repeated measures with treatment group, visit, treatment-by-visit interaction, and stratification factors (newly diagnosed or relapsed GPA or MPA, proteinase 3- or myeloperoxidase-ANCA, and rituximab or cyclophosphamide) as covariates. All statistical analyses were performed using Statistical Analysis System (SAS) software (V.9.4 of SAS for Windows, SAS Institute).

## Results

### Patients

In the ADVOCATE trial, 331 patients were randomized; however, 1 did not receive study medication. Of the 330 patients who received study medication, 268 (81.2%) had kidney involvement at baseline and were included in this kidney subgroup analysis (avacopan group, 134; prednisone taper group, 134).

Most baseline clinical characteristics of the subgroup were similar between the 2 treatment groups (Table 1) and did not differ from those of the entire ADVOCATE study population.^14^ Mean (SD) age was 61.5 (14.2) years; 59.7% of patients were male and 84.3% were White. Though balanced between treatment groups, there were more patients positive for MPO-ANCA (*n* = 168) than PR3-ANCA (*n* = 100), most patients were newly diagnosed (avacopan, 73.1%; prednisone taper, 74.6%), and most patients received induction treatment with rituximab (60.8% [163/268]). Of the 268 patients with kidney involvement, 265 had available baseline eGFR data. Mean (SD) baseline eGFR was 44.6 (27.7) ml/min per 1.73 m^2^ in the avacopan group and 45.6 (27.3) ml/min per 1.73 m^2^ in the prednisone taper group. One patient with a baseline eGFR < 15 ml/min per 1.73 m^2^ was enrolled in each treatment group (eligibility deviation). Mean (SD) UACR was higher in the avacopan group than in the prednisone taper group (815.8 [1035.6] mg/g vs. 639.7 [829.2] mg/g); the percentage of patients with UACR > 300 mg/g creatinine^20^ at baseline was higher in the avacopan group than in the prednisone taper group (60.4% [81/134 patients] vs. 55.2% [74/134]).

**Table 1:** Demographic and baseline clinical characteristics for patients with kidney involvement

Characteristic	Prednisone taper, *n* = 134	Avacopan, *n* = 134
Age, mean ± SD, yrs	62.2 ± 13.9	60.9 ± 14.6
Sex, *n* (%)		
Male	76 (56.7)	84 (62.7)
Female	58 (43.3)	50 (37.3)
Vasculitis disease status, *n* (%)		
Newly diagnosed	100 (74.6)	98 (73.1)
Relapsed	34 (25.4)	36 (26.9)
ANCA positivity status, *n* (%)		
Proteinase 3	47 (35.1)	53 (39.6)
Myeloperoxidase	87 (64.9)	81 (60.4)
Vasculitis type, *n* (%)		
GPA	63 (47.0)	65 (48.5)
MPA	71 (53.0)	69 (51.5)
BVAS^a^, mean ± SD	17.5 ± 5.2	17.4 ± 5.8
BVAS organ involvement, *n* (%)		
General	93 (69.4)	83 (61.9)
Ear, nose, and/or throat	44 (32.8)	49 (36.6)
Chest/lung	55 (41.0)	49 (36.6)
Mucous membranes/eyes	32 (23.9)	19 (14.2)
Cutaneous	17 (12.7)	22 (16.4)
Nervous system	28 (20.9)	31 (23.1)
Abdominal	0 (0)	4 (3.0)
Cardiovascular	3 (2.2)	3 (2.2)
Kidney manifestation of vasculitis on the BVAS^b^, *n* (%)		
RBC casts and/or glomerulonephritis	61 (45.5)	63 (47.0)
Hypertension	23 (17.2)	20 (14.9)
Proteinuria > 1+ or > 0.2 g/g creatinine	106 (79.1)	109 (81.3)
Hematuria ≥ 10 RBCs/hpf	72 (53.7)	79 (59.0)
Serum creatinine 125–249 μmol/l	62 (46.3)	59 (44.0)
Serum creatinine 250–499 μmol/l	19 (14.2)	27 (20.1)
Serum creatinine ≥ 500 μmol/l	0 (0.0)	1 (0.7)
Rise in serum creatinine > 30% or fall in creatinine clearance > 25%	20 (14.9)	18 (13.4)
Other	0 (0.0)	1 (0.7)
eGFR (ml/min per 1.73 m^2^), mean ± SD (range) (*N*1)	45.6 ± 27.3 (12.0–140.0) (134)	44.6 ± 27.7 (14.0–117.0) (131)
eGFR category, *n* (%)		
eGFR ≥ 90	10 (7.5)	17 (12.7)
eGFR 60–89	25 (18.7)	16 (11.9)
eGFR 45–59	21 (15.7)	14 (10.4)
eGFR 30–44	30 (22.4)	32 (23.9)
eGFR 15–29	47 (35.1)	51 (38.1)
eGFR < 15^c^	1 (0.7)	1 (0.7)
Unknown	0 (0.0)	3 (2.2)
Hematuria, *n* (%)		
< 10 RBCs/hpf	38 (28.4)	33 (24.6)
≥ 10 RBCs/hpf	92 (68.7)	97 (72.4)
Not assessed	4 (3.0)	4 (3.0)
UACR (mg/g), mean ± SD (range) (number of patients evaluated)	639.7 ± 829.2 (4.0–5367.0) (131)	815.8 ± 1035.6 (5.0–6461.0) (128)
UACR categories (mg/g), *n* (%)		
< 10	3 (2.2)	3 (2.2)
10–300	54 (40.3)	44 (32.8)
> 300	74 (55.2)	81 (60.4)
Not assessed	3 (2.2)	6 (4.5)
SF-36^d^, mean ± SD (number of patients evaluated)		
Physical Component Summary	40.4 ± 10.3 (130)	39.5 ± 10.7 (133)
Mental Component Summary	42.0 ± 13.4 (130)	44.5 ± 12.8 (134)
Immunosuppressant use		
i.v. rituximab	82 (61.2)	81 (60.4)
i.v. or oral cyclophosphamide	52 (38.8)	53 (39.6)

ANCA, antineutrophil cytoplasmic antibody; BVAS, Birmingham Vasculitis Activity Score; eGFR, estimated glomerular filtration rate; GPA, granulomatosis with polyangiitis; hpf, high powered field; MPA, microscopic polyangiitis; *N*1, number of patients evaluated; RBC, red blood cell; SF-36, 36-Item Short Form Health Survey; UACR, urine albumin-to-creatinine ratio.

a The BVAS is a composite measure of signs and symptoms in 9 organ systems. Scores range from 0 to 63, with higher scores indicating more extensive disease activity.

b As assessed by the adjudication committee.

c Patients included with eGFR < 15 were eligibility deviation.

d SF-36 Physical Component Summary and Mental Component Summary scores range from 0 to 100, with higher scores indicating better quality of life.

### Efficacy

#### Remission

**Figure 1:**
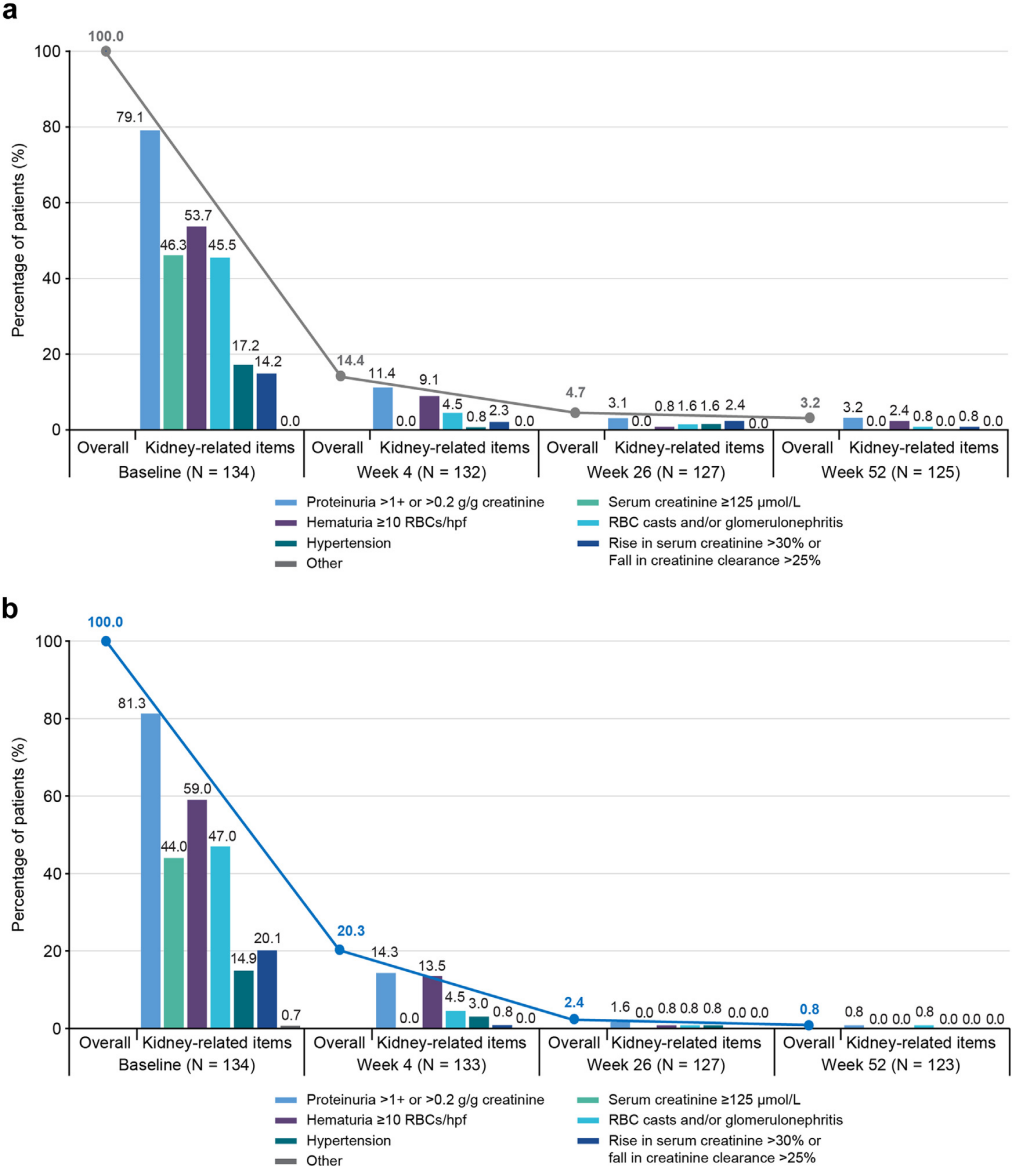
Percentage of patients with active kidney manifestations over time for (a) prednisone taper and (b) avacopan. hpf, high powered field; RBC, red blood cell.

Remission at week 26 was observed in 99 of 134 patients (73.9%) in the avacopan group and 95 of 134 patients (70.9%) in the prednisone taper group (estimated common difference [95% CI]: 3.0% [−7.7% to 13.7%]) (Table 2). Across eGFR categories, patients in both treatment groups achieved remission, with numerically more in the avacopan group, except for the eGFR ≥ 90 ml/min per 1.73 m^2^ subcategory. Sustained remission at week 52 was observed in 91 of 134 patients (67.9%) in the avacopan group and 76 of 134 patients (56.7%) in the prednisone taper group (estimated common difference [95% CI]: 11.2% [−0.3% to 22.7%]). Across eGFR categories, sustained remission rates were higher in the avacopan group except in the eGFR ≥ 90 ml/min per 1.73 m^2^ subgroup (Table 2). At weeks 4, 26, and 52, the percentages of patients with active kidney manifestations were 20.3%, 2.4%, and 0.8%, respectively, in the avacopan group; and 14.4%, 4.7%, and 3.2%, respectively, in the prednisone taper group (Figure 1a, b; Supplementary Table S1).

**Table 2:** Rates of remission, sustained remission, relapse, and change from baseline to week 52 in eGFR in patients with kidney involvement at baseline

Outcome	Prednisone taper *n* = 134	Avacopan *n* = 134	Difference (95% CI) or HR (95% CI)^a^
Remission at week 26^b^, *n* (%)	95 (70.9)	99 (73.9)	3.0 (−7.7 to 13.7)
Stratified by baseline eGFR category (ml/min per 1.73 m^2^), *n*/*N*1 (%)			
eGFR ≥ 90	7/10 (70.0)	11/17 (64.7)	−5.3 (−41.7 to 31.1)
eGFR 60–89	17/25 (68.0)	13/16 (81.3)	13.3 (−13.2 to 39.7)
eGFR 45–59	16/21 (76.2)	12/14 (85.7)	9.5 (−16.3 to 35.4)
eGFR 30–44	22/30 (73.3)	24/32 (75.0)	1.7 (−20.1 to 23.5)
eGFR 15–29	32/47 (68.1)	36/51 (70.6)	2.5 (−15.8 to 20.8)
Sustained remission at week 52^c^, *n* (%)	76 (56.7)	91 (67.9)	11.2 (−0.3 to 22.7)
Stratified by baseline eGFR category (ml/min per 1.73 m^2^), *n*/*N*1 (%)			
eGFR ≥ 90	6/10 (60.0)	9/17 (52.9)	−7.1 (−45.6 to 31.5)
eGFR 60–89	12/25 (48.0)	11/16 (68.8)	20.8 (−9.2 to 50.7)
eGFR 45–59	13/21 (61.9)	10/14 (71.4)	9.5 (−22.0 to 41.0)
eGFR 30–44	16/30 (53.3)	23/32 (71.9)	18.5 (−5.2 to 42.2)
eGFR 15–29	28/47 (59.6)	35/51 (68.6)	9.1 (−9.9 to 28.0)
Relapse after BVAS = 0 achieved at any time, *n*/*N*1 (%)	27/129 (20.9)	12/128 (9.4)	0.43 (0.22–0.85)
Kidney relapse^d^, *n*/*N*1	16/128 (12.5)	9/126 (7.1)	0.54 (0.24–1.23)
Change from baseline to week 52 in eGFR^e^ (ml/min per 1.73 m^2^)			
All patients with kidney involvement, LS mean ± SEM^d^ [*N*2]	4.1 ± 1.0 (125)	7.3 ± 1.1 (119)	3.2 (0.3–6.1)
eGFR ≥ 90	−19.1 (7.3) (10)	−11.7 (−21.8 to −1.6) (17)	7.4 (−11.5 to 26.3)
eGFR 60–89	−0.1 (2.3) (25)	−2.6 (3.0) (16)	−1.6 (−9.1 to 5.9)
eGFR 45–59	2.6 (2.2) (21)	9.4 (2.9) (14)	6.8 (−0.5 to 14.0)
eGFR 30–44	11.2 (1.8) (30)	11.7 (1.8) (32)	0.6 (−4.5 to 5.6)
eGFR 15–29	7.8 (1.4) (47)	13.5 (1.4) (51)	5.6 (1.7–9.5)
Patients who had week 26 urinalysis ≥ 10 RBC/hpf, LS mean (95% CI) (*N*2)	−2.0 (−8.3 to 4.2) (20)	8.6 (2.6–14.6) (18)	10.7 (1.9–19.4)
Patients with a baseline BVAS > 0 in the rise in creatine > 30% or fall in creatinine clearance > 25% item, LS mean (95% CI) (*N*2)	8.2 (2.7–13.6) (20)	19.2 (13.6–24.9) (18]	11.1 (3.2–18.9)

BVAS, Birmingham Vasculitis Activity Score; CI, confidence interval; eGFR, estimated glomerular filtration rate; GPA, granulomatosis with polyangiitis; hpf, high powered field; HR, hazard ratio; LS, least squares; MMRM, mixed-effects models for repeated measures; MPA, microscopic polyangiitis; *N*1, number of patients with observed data; *N*2, number of patients evaluated; RBC, red blood cell.

a For remission, CIs for treatment proportions were calculated using the Clopper-Pearson method. Two-sided 95% CIs were calculated for the difference in proportions (avacopan minus prednisone) using the Wald method. For relapse/kidney relapse, the Cox proportional hazard model was used to estimate the HR (95% CI).

b Remission was defined as a BVAS of 0 and no receipt of glucocorticoids for the treatment of GPA or MPA 4 weeks before week 26.

c Sustained remission was defined as remission at week 26 and a BVAS of 0 at week 52 with no receipt of glucocorticoids for the treatment of GPA or MPA 4 weeks before week 52, and no relapse between weeks 26 and 52.

d Kidney relapse was defined as time to first recurrence of a kidney manifestation of vasculitis (per the BVAS) after kidney manifestations of vasculitis had resolved, irrespective of any clinical intervention.

e For changes from baseline, LS mean and SEM were calculated from MMRM with treatment group, visit, and treatment-by-visit interaction as factors and baseline as a covariate.

#### Relapse

The relapse rate after remission at any time was 9.4% (12/128 patients) in the avacopan group compared with 20.9% (27/129 patients) in the prednisone taper group (hazard ratio [95% CI]: 0.43 [0.22–0.85]), for a relapse reduction risk of 57% (Table 2). The rate of kidney relapse was 7.1% (9/126 patients) in the avacopan group compared with 12.5% (16/128 patients) in the prednisone taper group (hazard ratio [95% CI]: 0.54 [0.24–1.23]), with a risk reduction of 46% (Table 2).

Eight patients achieved remission at week 26, but experienced a kidney relapse after remission. Of these 8 patients, 3 were in the avacopan group and 5 were in the prednisone taper group (Supplementary Table S2). The kidney manifestations were hypertension only for 1 patient; the other 7 patients had at least hematuria and/or proteinuria. Five of 8 patients (62.5%) had UACR > 300 mg/g at week 26. In addition, 5 of the 8 patients (62.5%) had ≥ 10 RBCs/hpf on urinalysis at week 26.

One hundred eighty-six patients achieved remission at week 26 and did not experience a kidney relapse after remission. Twenty of the 130 patients with available urinalysis data (15.4%) had ≥ 10 RBCs/hpf at week 26. Thus, most patients who achieved remission and subsequently did not have a kidney relapse after remission had < 10 RBCs/hpf on urinalysis at week 26, whereas most of those who did have a kidney relapse after remission had ≥ 10 RBCs/hpf.

#### Change in eGFR Over Time

eGFR improved from baseline in patients with kidney involvement in both treatment groups (Table 2). As previously reported, for all patients with kidney involvement at baseline, LS mean (SEM) increase in eGFR at week 52 was 7.3 (1.1) ml/min per 1.73 m^2^ in the avacopan group and 4.1 (1.0) ml/min per 1.73 m^2^ in the prednisone taper group. By eGFR categories, increases from baseline in eGFR over 52 weeks were observed for both treatment groups for eGFR categories of 45 to 59, 30 to 44, and 15 to 29 ml/min per 1.73 m^2^. The largest LS mean difference between the avacopan and prednisone taper groups in eGFR among these eGFR categories was in the eGFR category of 45 to 59 ml/min per 1.73 m^2^ (6.8), followed by the eGFR category of 15 to 29 ml/min per 1.73 m^3^ (5.6) (Table 2).

**Figure 2:**
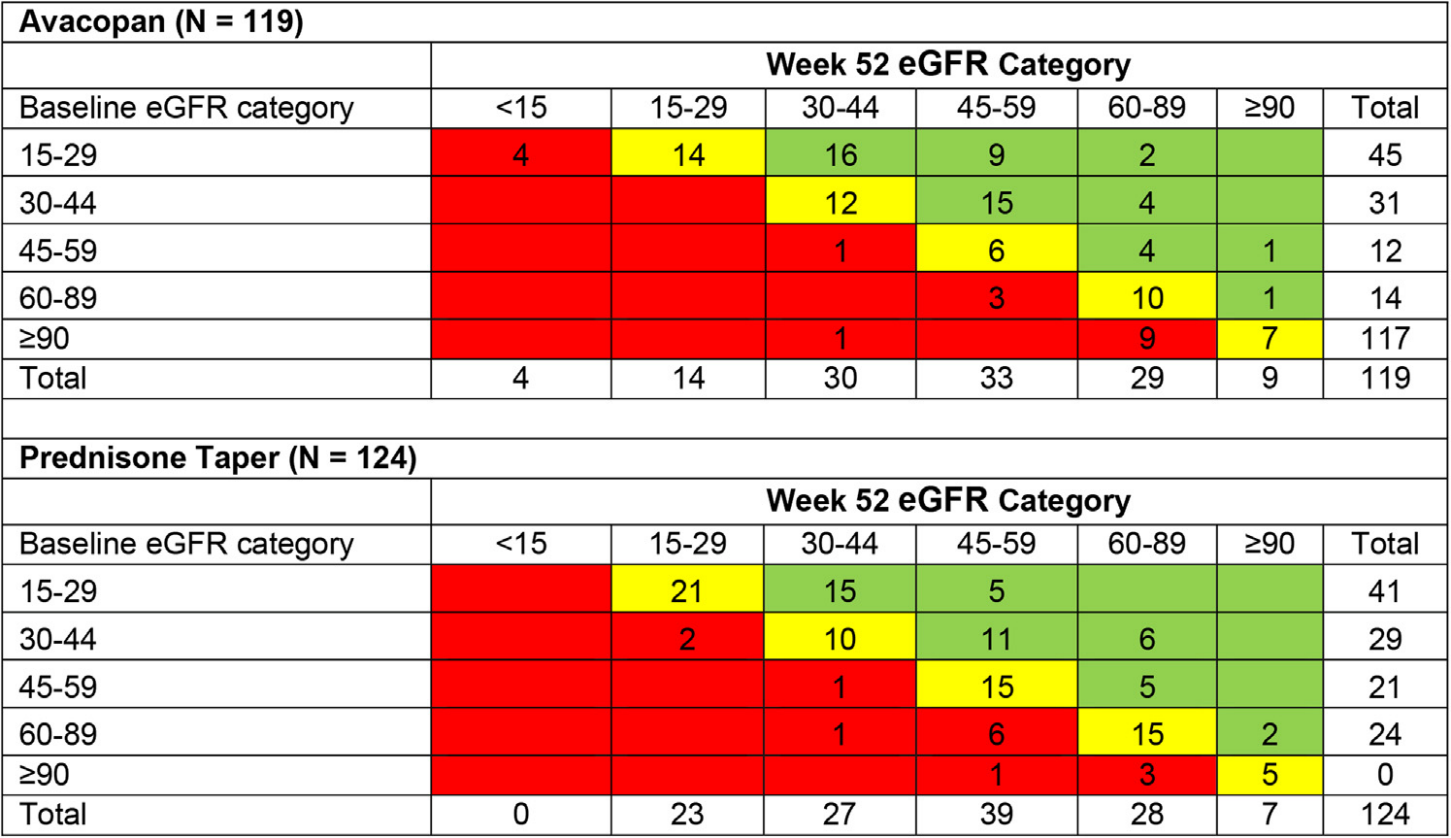
Change in eGFR category between baseline and week 52 in patients with eGFR ≥ 15 ml/min per 1.73 m^2^ at baseline. *N* includes patients with baseline eGFR ≥ 15 ml/min per 1.73 m^2^ and an eGFR assessment at week 52. Red: patients who had a worse eGFR category at week 52 compared with eGFR category at baseline. Yellow: patients who had the same eGFR category at week 52 as baseline. Green: patients who had an improved eGFR category at week 52 compared with eGFR category at baseline. eGFR, estimated glomerular filtration rate.

In the avacopan group, 119 of 134 patients (88.8%) had baseline eGFR data, week 52 eGFR data, and a baseline eGFR ≥ 15 ml/min per 1.73 m^2^ (Figure 2). Of these, 49 patients (41.2%) remained in the same eGFR category, 52 (43.7%) improved by ≥ 1 eGFR category, and 18 (15.1%) worsened by ≥ 1 eGFR category. In the prednisone group, 124 of 134 patients (92.5%) had baseline and week 52 eGFR data and a baseline eGFR ≥ 15 ml/min per 1.73 m^2^. Of these, 67 (54.0%) remained in the same eGFR category, 43 patients (34.7%) improved by ≥ 1 eGFR category, and 14 patients (11.3%) worsened by ≥ 1 eGFR category.

#### Change in Albuminuria Over Time

**Figure 3:**
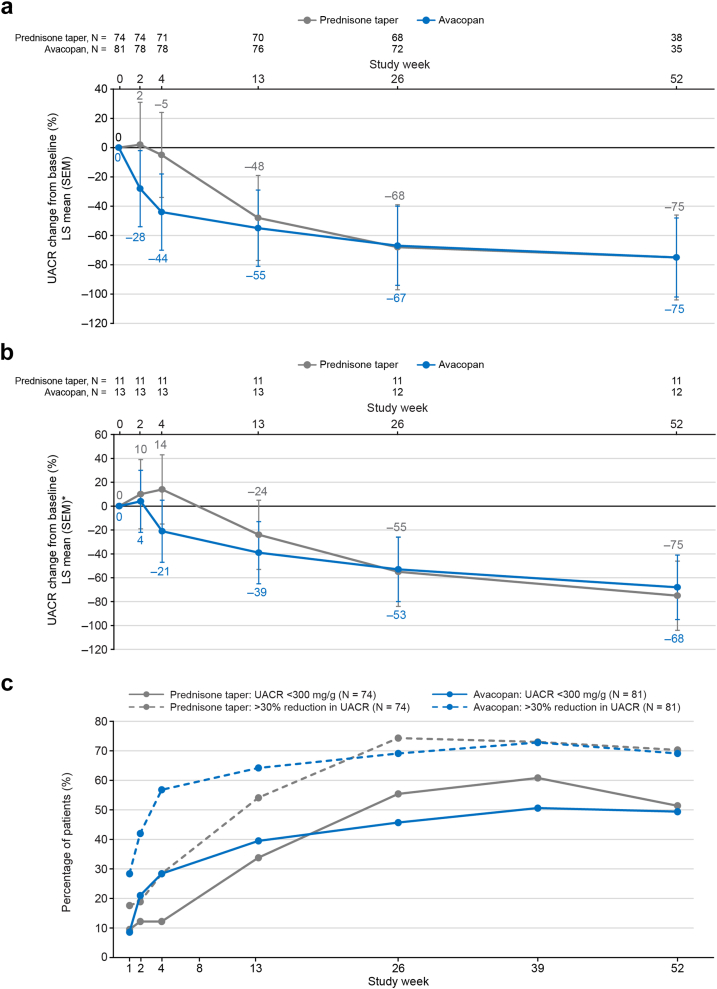
Change in UACR in patients with kidney involvement. (a) Change in UACR from baseline in patients with kidney involvement and baseline UACR > 300 mg/g. (b) Change in UACR from baseline in patients with baseline UACR > 300 mg/g and baseline adjudicated BVAS item rise in serum creatinine > 30% or fall in creatinine clearance > 25%. (c) Percentage of patients achieving either a UACR < 300 mg/g or a > 30% reduction in albuminuria. ^∗^Imprecise estimations because of the limited patient number. LS mean and SEM calculated from mixed effects models for repeated measures with treatment group, visit, and treatment-by-visit interaction as factors; and baseline as a covariate. Logarithmic transformations were applied to the data before fitting the model. Percent changes from baseline are based on ratios of geometric means of visit over baseline. CI, confidence interval; BVAS, Birmingham Vasculitis Activity Score; eGFR, estimated glomerular filtration rate; LS, least squares; UACR, urinary albumin-to-creatinine ratio.

In patients with kidney involvement and UACR > 300 mg/g creatinine at baseline, UACR improved more rapidly in the avacopan group than in the prednisone taper group (Figure 3a, Supplementary Table S3). At week 4, the LS mean change in UACR was −44% in the avacopan group compared with −5% in the prednisone taper group; the LS mean (95% CI) difference between treatment groups was −41% (−56% to −20%). By week 26, the decreases in UACR were similar in both treatment groups. Similarly, across all baseline eGFR categories, reduction in UACR was observed sooner for patients in the avacopan group than for those in the prednisone group, with similar reductions for the treatment groups observed by week 26 (Supplementary Table S3).

In patients with baseline UACR > 300 mg/g and baseline adjudicated BVAS item “rise in serum creatinine > 30% or fall in creatinine clearance > 25%,” improvement in UACR occurred more rapidly in the avacopan group than in the prednisone taper group (Figure 3b, Supplementary Table S3). The LS mean (95% CI) difference between the avacopan group and the prednisone taper group was −30% (−65% to −39%) at week 4. This indicates that proteinuria improves even for patients who show evidence of rapidly worsening kidney function at presentation. In patients with baseline UACR > 300 mg/g, achievement of either a UACR < 300 mg/g or a > 30% reduction in albuminuria presented earlier in the avacopan group than in the prednisone taper group (Figure 3c, Supplementary Table S3).

#### Change in Hematuria Over Time

**Figure 4:**
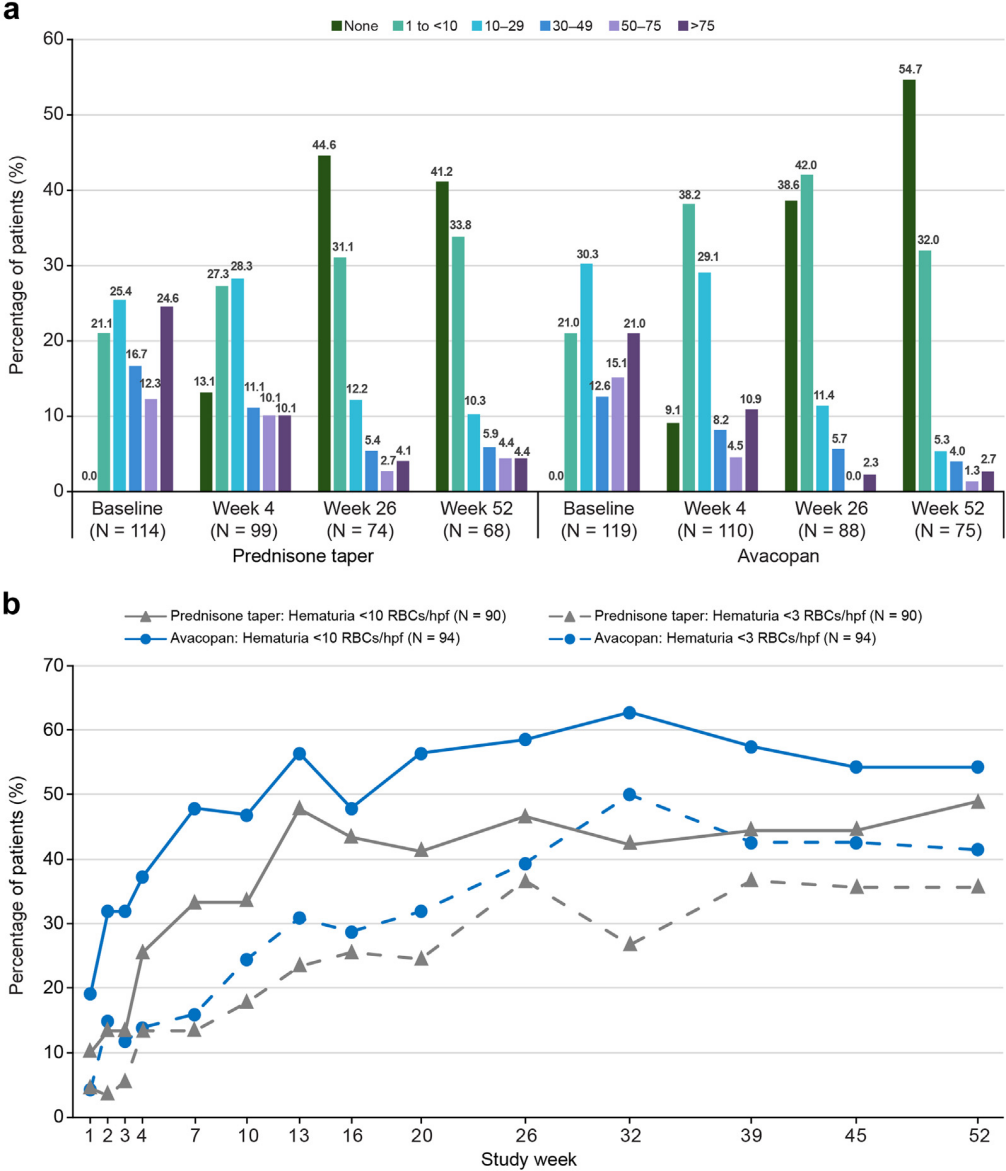
Changes in hematuria in patients with kidney involvement. (a) Percentage of patients with kidney involvement and hematuria at baseline by hematuria categories of none, 1 to < 10, 10 to 29, 30 to 49, 50 to 75, > 75 RBCs/hpf. (b) Percentage of patients with baseline hematuria ≥ 10 RBCs/hpf who achieved urinary RBC count < 10/hpf or < 3/hpf at any time during the 52 weeks. Missing central laboratory urinalysis was imputed as no achievement. hpf, high powered field; RBC, red blood cell.

In patients with kidney involvement and hematuria (at least 1–2 RBCs/hpf) at baseline, hematuria decreased over time in both treatment groups (Figure 4a). In patients with baseline hematuria ≥ 10 RBCs/hpf, the percentage of patients achieving hematuria < 10 RBCs/hpf or < 3 RBCs/hpf during the 52-week treatment period increased in both treatment groups, with more patients in the avacopan group achieving these reductions at earlier timepoints than those in the prednisone taper group (Figure 4b).

#### GC Use

The total GC use over 52 weeks was lower in the avacopan group than in the prednisone taper group (Supplementary Table S4). From weeks 0 to 4, the percentage of patients who received GCs was 80.6% (108/134) in the avacopan group and 100% (134/134) in the prednisone taper group. During that time (weeks 0–4), mean total prednisone-equivalent dose of all oral and i.v. GCs was 598 mg in the avacopan group and 1612 mg in the prednisone taper group. From weeks 0 to 26, the percentage of patients who received GCs was 84.3% (113/134) in the avacopan group and 100% (134/134) in the prednisone taper group. During that time (weeks 0–26), mean total prednisone-equivalent dose of all oral and i.v. GCs was 1276 mg in the avacopan group and 3319 mg in the prednisone taper group. Similar percentages of patients received i.v. GCs (avacopan, 67.9% [91/134 patients]; prednisone taper, 70.1% [94/134 patients]) from week 0 to 26. From weeks 27 to 52, mean total prednisone-equivalent dose of all oral and i.v. GCs was 333 mg in the avacopan group and 505 mg in the prednisone taper group. Similar percentages of patients received non–study-supplied i.v. GCs (avacopan, 68.7% [92/134 patients]; prednisone taper, 72.4% [97/134 patients]) from weeks 0 to 52. Mean non–study-supplied prednisone-equivalent dose received from weeks 0 to 52 was similar in both groups (avacopan 1589 mg; prednisone taper 1416 mg).

#### GC Toxicity

GC-induced toxicity, as assessed by the GTI cumulative worsening score and GTI aggregate improvement score at week 26, was greater in the prednisone taper group than in the avacopan group, with LS mean (95% CI) difference of −19.6 (−29.6 to −9.6) and −13.1 (−23.2 to −3.0), respectively (Supplementary Table S4).

#### HRQoL

HRQoL as evaluated by the SF-36 for Physical Component Summary Score and Mental Component Summary Score tended to improve with both treatments, with greater improvements observed in the avacopan group than in the prednisone taper group, with LS mean change from baseline (95% CI) difference of 1.8 (−0.3 to 4.0) and 1.5 (−1.0 to 4.0), respectively (Supplementary Table S5).

### Safety

Serious AEs occurred in 61 of 134 patients (45.5%; 104 events) in the avacopan group and 65 of 134 patients (48.5%; 148 events) in the prednisone taper group (Supplementary Table S6). Serious infections occurred in 20 of 134 patients (14.9%; 23 events) in the avacopan group and 24 of 134 patients (17.9%; 30 events) in the prednisone taper group. Serious AEs of abnormality on liver function testing occurred in 8 of 134 patients (6.0%) in the avacopan group and 5 of 134 patients (3.7%) in the prednisone taper group. Eight patients required temporary or permanent dialysis. Three patients in the avacopan group received dialysis (permanent in 2 patients and a single session in 1 patient) and dialysis was planned in 1 additional patient (expected to be permanent); 4 patients in the prednisone taper group received dialysis (permanent in 2 patients, temporary in 1 patient, and additional information was not available for 1 patient whose eGFR was 7 ml/min per 1.73 m^2^ at their study end [day 184]). The AEs leading to death for the 3 patients who died in the prednisone taper arm were as follows: (i) diarrhea, vomiting, and fungal infection; (ii) infectious pleural effusion; and (iii) death of unknown cause. AEs leading to death for the 2 patients in the avacopan arm were as follows: (i) GPA and (ii) pneumonia. Avacopan was stopped before death for both patients who died in the avacopan arm (on day 236 for the patient who died on day 315, and on day 50 for the patient who died on day 160).

## Discussion

ADVOCATE compared outcomes in patients treated with background immunosuppressive therapy combined with either avacopan for 52 weeks or prednisone administered on a tapering schedule over 20 weeks.^14^ Analysis of data from the ADVOCATE subgroup of patients with GPA or MPA with kidney involvement at baseline demonstrated similar results for the avacopan group and the prednisone taper group in achieving remission at week 26, and a higher rate of sustained remission at week 52 for the avacopan group, aligning with the results from the entire ADVOCATE study.^14^ Further, this analysis showed that across eGFR categories ≥ 15 ml/min per 1.73 m^2^, patients in both treatment arms achieved remission and sustained remission with results favoring the avacopan group except at an eGFR ≥ 90 ml/min per 1.73 m^2^, where the number of patients was fewer than in any other eGFR subcategory. Relapse rates after remission were lower in the avacopan group than in the prednisone taper group. Given the *post hoc* nature of this analysis, no statistical analysis of treatment differences was performed.

In addition to the efficacy outcomes of remission and relapse rates, other outcomes reported in this study favored the avacopan group, including trends of kidney recovery, faster reduction in proteinuria and hematuria, reduction in GC-related toxicity, and improvement in HRQoL outcomes.

Although avacopan initially led to a faster reduction in albuminuria during the first month, this difference diminished after week 13, resulting in similar rates of albuminuria between the treatment groups at weeks 26 and 52. Because most patients in the avacopan group received GCs during the screening period and first weeks of the trial, it would be interesting to understand the potential additional effect of avacopan and GCs. In the 12-week CLEAR phase 2 study,^25^ outcomes from patients receiving avacopan plus a 20 mg prednisone taper were compared with those from patients receiving avacopan without a prednisone taper. At week 4, the reduction in albuminuria was 40% for the patients receiving prednisone taper and 47% for those not receiving a prednisone taper.^25^ Additional studies with a larger sample size are required to more completely understand the impact of avacopan on albuminuria. Furthermore, although proteinuria at 6 months is associated with long-term kidney and survival outcome in ANCA-associated vasculitis,^26^^,^^27^ data are lacking regarding the potential prognostic role of the speed of reduction of proteinuria during the first weeks of treatment.

Considering that avacopan is not known to affect intraglomerular hemodynamics, taking together the eGFR changes, UACR reductions (including in patients with acutely worsening eGFR), and rates of patients achieving hematuria < 10 RBCs/hpf or < 3 RBCs/hpf for patients in the avacopan group, these data suggest that an immunologic phenomenon is involved with a rapid, tissue-targeted control of neutrophil activation and inflammation leading to improvement in glomerular pathology and permeability.

Studies in patients with kidney involvement in ANCA-associated vasculitis (GPA, MPA, or renal-limited vasculitis)^27^, ^28^, ^29^ described that the persistence of hematuria may be a sign of low-level disease activity or risk for potential kidney relapse. Though not evaluated by treatment group because of small numbers, with 62.5% of patients with a kidney relapse after remission having hematuria > 10 RBCs/hpf at 26 weeks compared with 15.4% of patients who did not have a kidney relapse after remission, this study confirms the possibility that the presence of hematuria > 10 RBCs/hpf may predict a future clinical kidney relapse for patients with GPA or MPA.

Higher GC use was observed in the prednisone taper group, with 37% being non–study-supplied GC. However, non–study-supplied GC use was similar in both groups, indicating that use of avacopan did not increase the need for non–study-supplied GCs. According to data from trials in vasculitis, treatment-related damage occurs secondary to the use of GCs, and higher levels of damage are independently associated with the duration of GC use.^13^ Two trials for induction of remission in ANCA-associated vasculitis (GPA or MPA) demonstrated that a reduced-dose GC regimen was noninferior to a high-dose GC regimen.^30^^,^^31^ The results in the ADVOCATE study suggest that patients treated with avacopan may be able to achieve and sustain remission using GC doses even lower than previously described.^30^ Although a reduction in GC-related toxicity was observed in the avacopan group compared with the prednisone taper group, the patient incidence of serious AEs was similar between the treatment groups. However, the total number of serious AEs experienced in the avacopan group was lower compared to that in the prednisone taper group.

HRQoL results described here, as evaluated by the SF-36, were similar to those of the overall trial^14^^,^^32^^,^^33^ and consistent with findings from previous trials,^25^^,^^34^ with greater improvements observed in the avacopan group than in the prednisone taper group.

The strengths of this study include the involvement of a large cohort of patients with GPA or MPA recruited into a clinical trial from 143 centers internationally, with the trial cohort representative of other trial populations in GPA and MPA. In addition, there was a rigorous study design and analysis with minimal loss to follow-up.

However, this study also has some limitations to consider. Patients with eGFR < 15 ml/min per 1.73 m^2^ and those with alveolar hemorrhage requiring mechanical ventilation were not included in the trial (except 2 patients enrolled with eligibility deviations due to eGFR), and findings of efficacy and safety of avacopan in patients with kidney involvement need to be confirmed in this population. The information presented is based on a subgroup analysis with a limited number of patients in the treatment groups and when describing data by eGFR category the number of patients becomes even lower; thus, only descriptive evaluations are presented. In addition, there is limited data on the use of avacopan beyond 52 weeks. Longer follow-up is required to more fully understand the benefits and risks of the use of avacopan therapy for GPA or MPA. Data concerning blood pressure levels and use of angiotensin-converting enzyme inhibitors, angiotensin receptor blockers, or sodium-glucose cotransporter-2 inhibitors were not available for this analysis. The impact of preexisting kidney disease, diabetes, or hypertension was also not evaluated in this study. Furthermore, although repeat dosing of rituximab is the currently recommended treatment approach for GPA and MPA,^35^, ^36^, ^37^ the approved treatment at the time of the ADVOCATE trial was not to administer repeat rituximab at week 26.^32^ Thus, the efficacy and safety of avacopan when used alongside rituximab for maintenance of remission is unknown.

In conclusion, similar to the results of the overall ADVOCATE trial, those of the current subgroup analysis suggest that in patients with GPA or MPA and kidney involvement at baseline, treatment with avacopan, compared with treatment with a prednisone taper, achieved similar rates of improved recovery of kidney function, led to faster reduction in albuminuria and hematuria, and lowered GC-related toxicity, with an acceptable safety profile.

## Appendix

### List of the members of the ADVOCATE Study Group

(Presented by country, with National Coordinating Center followed by participating centers, in alphabetical order by principal investigator.)

**Australia**—National Coordinating Center: Royal Adelaide Hospital, Adelaide, SA (C. Au Peh); Sir Charles Gairdner Hospital, Nedlands, Western Australia (A. Chakera); Royal North Shore Hospital, St Leonards (B. Cooper); Griffith University, Southport (J. Kurtkoti); Wesley Medical Research, Auchenflower (D. Langguth); Western Health, St. Albans, Victoria (V. Levidiotis); Prince of Wales Hospital, Randwick, New South Wales (G. Luxton); Austin Health, Heidelberg, Victoria (P. Mount); Princess Alexandra Hospital, Woolloongabba, Queensland (D. Mudge); Sunshine Coast University Hospital, Birtinya (E. Noble); Westmead Hospital, Westmead, New South Wales (R. Phoon); Royal Brisbane and Women's Hospital, Herston, Queensland (D. Ranganathan); Concord Repatriation General Hospital, Concord (A. Ritchie); Monash Medical Centre, Clayton, Victoria (J. Ryan); Liverpool Hospital, Liverpool, New South Wales (M. Suranyi).

**Austria**—National Coordinating Center: Medizinische Universitaet Graz, Graz (A. Rosenkranz); Landeskrankenhaus Feldkirch, Feldkirch (K. Lhotta); Medical University of Innsbruck, Innsbruck (A. Kronbichler).

**Belgium**—National Coordinating Center: Cliniques Universitaires Saint-Luc, Brussels (N. Demoulin); Centre Hospitalier Universitaire (CHU) de Liege, Liege (C. Bovy); Antwerp University Hospital (UZA), Edegem (R. Hellemans); Université Libre de Bruxelles (ULB) - Hôpital Erasme, Brussels (J. Hougardy); University Hospital (UZ) Leuven, Leuven (B. Sprangers); University Hospital Brussels, Brussels (K. Wissing).

**Canada**—National Coordinating Center: University of Toronto, Toronto (C. Pagnoux); St. Paul Hospital, Vancouver (S. Barbour); Centre de Recherche du Centre Hospitalier de l'Université de Montréal, Montreal (S. Brachemi); CISSS de la Monteregie-Centre – Hôpital Charles LeMoyne, Greenfield Park (S. Cournoyer); University of Calgary, Calgary (L. Girard); Hospital Maisonneuve-Rosemont, Montreal (L. Laurin); Centre Hospitalier Universitaire de Sherbrooke, Sherbrooke (P. Liang); CHUQ-L'Hotel-Dieu de Quebec, Quebec City (D. Philibert); St. Josephs Healthcare, Hamilton (M. Walsh).

**Czech Republic**—Department of Nephrology, General University Hospital, Prague (V. Tesar); Rheumatology Institute, Prague (R. Becvar); University Hospital Olomouc, Olomouc (P. Horak); University Hospital Vinohrady, Prague (I. Rychlik).

**Denmark**—National Coordinating Center: Copenhagen University Hospital, Copenhagen (W. Szpirt); Odense University Hospital, Odense (H. Dieperink); Aalborg University Hospital, Aalborg (J. Gregersen); Aarhus University Hospital - Skejby, Aarhus (P. Ivarsen); Herlev Hospital, Herlev (E. Krarup); Sjaellands Universitetshospital Roskilde, Roskilde (C. Lyngsoe).

**France**—National Coordinating Center: CHU Bordeaux - Hospital Pellegrin, Bordeaux (C. Rigothier); CHU Angers, Angers (J. Augusto); CHU Lyon- Hôpital Femme- Mere-Enfant, Bron (A. Belot); CHU de Toulouse - Hospital Rangueil, Toulouse (D. Chauveau); CHU de Brest - Hopital de la Cavale Blanche, Brest (D. Cornec); APHM - Hôpital de la Conception, Marseille (N. Jourde-Chiche); CHU de Caen, Caen (M. Ficheux); Hôpital Européen Georges Pompidou, Paris (A. Karras); Hopitaux Civils de Colmar, Colmar (A. Klein); Hopitaux Prives de Metz, Metz (F. Maurier); Centre Hospitalier Boulogne sur Mer, Boulogne sur Mer (R. Mesbah); CHU Nimes – Hopital Caremeau, Nimes (O. Moranne); CHU Nantes Medicine Interne, Nantes (A. Neel); Centre Hospitalier de Valenciennes, Valenciennes (T. Quemeneur); Hospital Pitie Salpetriere, Paris (D. Saadoun); Hôpital Cochin, Paris (B. Terrier); CHU de Grenoble, Grenoble Isere Cedex, (P. Zaoui).

**Germany**—National Coordinating Center: University Clinic Heidelberg, Heidelberg (M. Schaier); University Clinic Mannheim, Mannheim (U. Benck); Clinic of Ludwigshafen am Rhein, Ludwigshafen (R. Bergner); University Clinic Jena, Jena (M. Busch); University Clinic Aachen, Aachen (J. Floege); University Clinic Cologne, Cologne (F. Grundmann); Medizinische Hochschule Hannover, Hannover (H. Haller); Klinikum Fulda, Fulda (M. Haubitz); Medius Clinic Kirchheim, Kirchheim-unter-Teck (B. Hellmich); University Hospital Tuebingen, Tuebingen (J. Henes); Nephrological Center Villingen-Schwenningen, Villingen-Schwenningen (B. Hohenstein); University Clinic Carl Gustav Carus, Dresden (C. Hugo); Klinikum Bad Bramstedt GmbH, Bad Bramstedt (C. Iking-Konert and F. Arndt); Asklepios Kinik, Hamburg (T. Kubacki and I. Kotter); University Clinic Schleswig-Holstein, Luebeck (P. Lamprecht); University Clinic Leipzig, Leipzig (T. Lindner and J. Halbritter); Charité - Universitaetsmedizin Berlin, Berlin (H. Mehling); Universität München – Großhadern, Munich (U. Schönermarck); University Clinic Freiburg, Freiburg (N. Venhoff); University Clinic Munich, Munich (V. Vielhauer); University Clinic Essen, Essen (O. Witzke).

**Hungary**—Qualiclinic Kft, Budapest (I. Szombati); DEOEC Rheumatology Faculty, Debrecen (G. Szucs).

**Italy**—National Coordinating Center: IRCCS Azienda Ospedaliera Universitaria San Martino, Genova (G. Garibotto); ASST Santi Paolo e Carlo-Presidio Ospedale San Carlo, Milan (F. Alberici); Istituto Clinico Humanitas, Rozzano (E. Brunetta); IRCCS Ospedale San Raffaele, Milan (L. Dagna); Azienda Sanitaria Universitaria Integrata di Udine, Udine (S. De Vita); Azienda Ospedaliero-Universitaria Careggi, Florence (G. Emmi); AOU Ospedali Riuniti di Ancona, Torrette Ancona (A. Gabrielli); Azienda Ospedaliero Universitaria di Parma, Parma (L. Manenti); ASST di Monza-Ospedale San Gerardo, Monza (F. Pieruzzi); ASL Città di Torino - Ospedale San Giovanni Bosco, Torino (D. Roccatello); Azienda Unità Sanitaria Locale di Reggio Emilia, Reggio Emilia (C. Salvarani).

**Japan**—National Coordinating Investigator: Prof. M. Harigai, Tokyo Women’s Medical University, Tokyo; Kagawa University Hospital, Kagawa (H. Dobashi); Hokkaido University Hospital, Hokkaido (T. Atsumi); University of Miyazaki Hospital, Miyazaki (S. Fujimoto); Teikyo University Chiba Medical Center, Chiba (N. Hagino); National Hospital Organization Yokohama Medical Center, Yokohama (A. Ihata); Kyorin University Hospital, Tokyo (S. Kaname); Keio University Hospital, Tokyo (Y. Kaneko); Juntendo University Shizuoka Hospital, Shizuoka (A. Katagiri); Nagoya Medical Center, Aichi (M. Katayama); Yokohama City University Hospital, Kanagawa (Y. Kirino); National Hospital Organization Kanazawa Medical Center, Ishikawa (K. Kitagawa); Akita University Hospital, Akita City (A. Komatsuda); Teikyo University Hospital, Tokyo (H. Kono); Saitama Medical Center, Saitama (T. Kurasawa); National Hospital Organization Chiba East Hospital, Chiba (R. Matsumura); Saitama Medical University Hospital, Saitama (T. Mimura); Kobe University Hospital, Hyogo (A. Morinobu); Shimane University Hospital, Shimane (Y. Murakawa); Nagoya City University Hospital, Aichi (T. Naniwa); Toho University Omori Medical Center, Tokyo (T. Nanki); Hamamatsu University Hospital, Shizuoka (N. Ogawa); National Hospital Organization Tokyo Medical Center, Tokyo (H. Oshima); Okayama University Hospital, Okayama (K. Sada); Hiroshima University Hospital, Hiroshima (E. Sugiyama); Osaka Medical College Hospital, Osaka (T. Takeuchi); Toyama University Hospital, Toyama (H Taki); Juntendo University Hospital, Tokyo (N. Tamura); Tazuke Kofukai Medical Research Institute Kitano Hospital, Osaka (T. Tsukamoto); University of Tsukuba Hospital, Ibaraki (K. Yamagata); Okayama Saiseikai General Hospital, Okayama (M. Yamamura).

**The Netherlands**—Erasmus MC, Rotterdam (P. van Daele); Groningen Universitair Medisch Centrum, Groningen (A. Rutgers); Leids Universitair Medisch Centrum, Leiden (Y. Teng).

**New Zealand**—National Coordinating Center: Dunedin Hospital, Dunedin (R. Walker); Christchurch Clinical Studies Trust, Christchurch (I. Chua); Auckland City Hospital, Auckland (M. Collins); Waikato Hospital, Hamilton (K. Rabindranath); North Shore Hospital, Takpuna, Auckland (J. de Zoysa).

**Norway**—National Coordinating Center: Akershus Universitetssykehus, Nordbyhagen (M. Svensson); Oslo Universitessykkehus, Oslo (B. Grevbo); University Hospital of North Norway, Tromso (S. Kalstad).

**Republic of Ireland**—National Coordinating Center: Beaumont Hospital, Dublin (M. Little); Cork University Hospital, Cork (M. Clarkson); St. Vincent's University Hospital, Dublin (E. Molloy).

**Spain**—Hospital Vall D Hebron, Barcelona (I. Agraz Pamplona); Hospital Sant Joan de Deu, Barcelona (J. Anton); Hospital Universitario Infanta Sofia, San Sebastian de los Reyes, Madrid (V. Barrio Lucia); Hospital Da Costa, Burela (S. Ciggaran); Hospital Clinic Barcelona – Autoimmune Diseases Department, Barcelona (M. Cinta Cid); Fundacio Puigvert, Barcelona (M. Diaz Encarnacion); Hospital Universitari de Bellvitge, Barcelona (X. Fulladosa Oliveras); Hospital del Mar, Barcelona (M. Jose Soler); Hospital Germans Trias i Pujol, Badalona (H. Marco Rusinol); Hospital 12 de Octubre, Madrid (M. Praga); Hospital Clinic Barcelona, Barcelona (L. Quintana Porras); Hospital Universitari Arnau de Vilanova, Lleida (A. Segarra).

**Sweden**—National Coordinating Center: Karolinska University Hospital, Stockholm (A. Bruchfeld); Linköping University, Linköping (M. Segelmark); Uppsala University Hospital, Uppsala (I. Soveri); Örebro University Hospital, Örebro (E. Thomaidi); Skane University Hospital, Malmo (K. Westman).

**Switzerland**—National Coordinating Center: Kantonsspital St. Gallen, St. Gallen (T. Neumann); CHUV Lausanne, Lausanne (M. Burnier); University Hospital Basel, Basel (T. Daikeler); Hôpital Fribourgeois, Fribourg (J. Dudler); Immunologie- Zentrum Zürich, Zürich (T. Hauser); Universitätsspital Zürich, Zürich (H. Seeger); Inselspital, Universitätsspital Bern, Bern (B. Vogt).

**UK**—National Coordinating Center: Addenbrooke’s Hospital - Cambridge University Hospitals, Cambridge (D. Jayne); Leicester General Hospital, Leicester (J. Burton and R. Al Jayyousi); Leeds Childrens Hospital, Leeds (T. Amin); Leeds Teaching Hospitals NHS Trust, Leeds (J. Andrews); Freeman Hospital, Newcastle upon Tyne (L. Baines); Great Ormond Street Hospital for Children, London (P. Brogan); Southend University Hospital, Westcliff on Sea (B. Dasgupta); Kent and Canterbury Hospital, Canterbury, Kent (T. Doulton); Royal Berkshire Hospital, Reading, Berkshire (O. Flossmann); University Hospital of Wales, Cardiff (S. Griffin); Royal Liverpool University Hospital, Liverpool (J. Harper); University of Birmingham, Birmingham (L. Harper); University of Aberdeen, Aberdeen (D. Kidder); Russells Hall Hospital, Dudley (R. Klocke); Queens Medical Centre, Nottingham (P. Lanyon); Nuffield Orthopaedic Centre, Oxford (R. Luqmani); Whytemans Brae Hospital, Fife (J. McLaren); St Helier Hospital, Carshalton (D. Makanjuola); Alder Hey Children's NHS Foundation Trust, Liverpool (L. McCann); Basildon University Hospital, Basildon (A. Nandagudi and S. Selvan); Salford Royal NHS Foundation Trust Manchester, Salford (E. O'Riordan); University of Manchester, Manchester Royal Infirmary, Manchester (M. Patel); Queen Elizabeth University Hospital, Glasgow (R. Patel); Imperial College Healthcare NHS Trust, London (C. Pusey); The Royal London Hospital, London (R. Rajakariar); Bristol Royal Infirmary, Bristol (J. Robson); Guy’s and St Thomas’s NHS Foundation Trust, London (M. Robson); UCL Centre for Nephrology Royal Free, London (A. Salama); Royal Devon and Exeter Hospital, Exeter (L. Smyth); Raigmore Hospital, Inverness (J. Sznajd); Dorset County Hospital, Dorchester (J. Taylor).

**USA**—University of Pennsylvania, Philadelphia (P. Merkel and A. Sreih); Winthrop University Hospital, Mineola (E. Belilos); Columbia University Medical Center, New York (A. Bomback); Virginia Mason Medical Center, Seattle (J. Carlin); University of South Florida, Tampa (Y. Chang Chen Lin); University of North Carolina Hospitals, Chapel Hill (V. Derebail); MedStar Georgetown University Hospital, Washington (S. Dragoi); University of Chicago Medical Center Rheumatology, Chicago (A. Dua); Cedars-Sinai Medical Center, Los Angeles (L. Forbess); Johns Hopkins Bayview Medical Center, Baltimore (D. Geetha); University of Michigan, Ann Arbor (P. Gipson); Rhode Island Hospital, Providence (R. Gohh); Brookview Hills Research Associates, Winston-Salem (G. T. Greenwood); Indiana University Nephrology, Indianapolis (S. Hugenberg); Western Washington Arthritis Clinic, Bothell (R. Jimenez); Northwest Louisiana Nephrology, Shreveport (M. Kaskas); University of California, Los Angeles, Santa Monica (T. Kermani); Altoona Center for Clinical Research, Duncansville (A. Kivitz); University of Utah, Salt Lake City (C. Koening); Cleveland Clinic, Cleveland (C. Langford); Northwell Health, Great Neck (G. Marder); University of Kentucky Medical Center, Lexington (A. Mohamed); Boston University, Boston (P. Monach); Arizona Kidney Disease and Hypertension Center Flagstaff, Flagstaff (N. Neyra); Articularis Healthcare Group, Charleston (G. Niemer); Massachusetts General Hospital, Boston (J. Niles); East Carolina University, Greenville (R. Obi); Renal Disease Research Institute, Dallas (C. Owens); Washington University School of Medicine, St. Louis (D. Parks); Colorado Kidney Care, Denver (A. Podoll); Ohio State University, Columbus (B. Rovin); San Francisco General Hospital Dialysis Center, San Francisco (R. Sam); Rheumatology Associates of North Alabama, Huntsville (W. Shergy); Boise Kidney & Hypertension, PLLC – Meridian, Caldwell (A. Silva); Mayo Clinic - Division of Pulmonary & Critical Care Medicine, Rochester (U. Specks); Hospital for Special Surgery, New York (R. Spiera); University of Kansas Medical Center, Kansas City (J. Springer); University of Colorado Denver - School of Medicine, Aurora (C. Striebich); Arizona Arthritis & Rheumatology Research, Phoenix (A. Swarup); University of Minnesota, Minneapolis (S. Thakar); Emory University School of Medicine, Atlanta (A. Tiliakos); Arthritis, Autoimmune and Allergy LLC, Daytona Beach (Y. Tsai); University of Texas Health Sciences Center, Houston (D. Waguespack); Allegheny General Hospital, Pittsburgh (M. Chester Wasko).

## Disclosure

DG received consulting fees from Amgen, ChemoCentryx (a wholly owned subsidiary of Amgen), Aurinia, Otsuka, Calliditas Therapeutics, Vera Therapeutics, and GlaxoSmithKline (GSK). FBC received consulting and/or speaker fees from Amgen, Aurinia, Calliditas, and Climb Bio. AB received consultant and speaker fees from Alexion, AstraZeneca, Bayer, Boehringer-Ingelheim, CSL Vifor, Fresenius, Otsuka, and GSK; received payment for expert testimony from the Swedish National Board of Health and Welfare; was a data safety monitoring board member for Alexion, Boehringer-Ingelheim, and CSL Vifor; was an associate editor of CJK; and had leadership roles at the Immunonephrology Working Group (IWG) of the European Renal Association (both unpaid roles). AKr reports grants or contracting fees from CSL Vifor and Otsuka; consulting fees from Amgen, AstraZeneca, Boehringer-Ingelheim, CSL Vifor, Delta4, GSK, Milteny Biotec, Novartis, Novo Nordisk, Otsuka, Roche, Sobi, and Walden Biosciences; received support to attend meeting from AstraZeneca and Otsuka; and had a leadership role at IWG of European Renal Association. AKa received consultant and speaker fees from AstraZeneca, Boehringer-Ingelheim, CSL Vifor, Otsuka, Novartis, and GSK. GNN received consultant or speaker fees from Amgen. PAM received grant or research support from AbbVie, AstraZeneca, Boehringer-Ingelheim, Bristol Myers Squibb, Eicos, Electra, Forbius, Genentech/Roche, GSK, InflaRx, Neutrolis, and Takeda; received consulting fees from AbbVie, Alpine, Amgen, ArGenx, AstraZeneca, Boeringher-Ingelheim, Bristol Myers Squibb, CSL Behring, GSK, HiBio, iCell, InflaRx, Janssen, Kinevant, Kyverna, Metagenomia, Novartis, NS Pharma, and Q32; holds stock or stock options in Kyverna, Q32, and Sparrow; and received royalties from UpToDate. SB is an employee of Amgen Ltd and owns stock in Amgen Inc. AMB is an employee of Amgen Inc. and owns stock in Amgen Inc. DRWJ received grant or research support from CSL Vifor; received consulting fees from Alentis, Amgen, AstraZeneca, Novartis, Chinook, CSL Vifor, GSK, Hansa, Otsuka, CSL Vifor, and Roche; received honoraria from CSL Vifor; was a data safety monitory board member for Chook and GSK; and owns stock or stock options in Alentis and Aurinia.

## Supplementary Material

Supplementary File (PDF) **Suppl****ementary Methods. Table S1.** Percentage of patients with active kidney manifestations on the Birmingham Vasculitis Activity Score over time. **Table S2.** Characteristics of patients who experienced a kidney relapse after remission. **Table S3.** Change in albuminuria in patients with kidney involvement. **Table S4.** GC use and toxicity in patients with kidney involvement. **Table S5.** Changes in the physical component summary score and mental component summary score of the SF-36 in patients with renal involvement. **Table S6.** Safety in patients with kidney involvement. CONSORT Checklist.

## Footnotes

Supplementary File (PDF)
Supplementary Methods. **Table S1.** Percentage of patients with active kidney manifestations on the Birmingham Vasculitis Activity Score over time. **Table S2.** Characteristics of patients who experienced a kidney relapse after remission. **Table S3.** Change in albuminuria in patients with kidney involvement. **Table S4.** GC use and toxicity in patients with kidney involvement. **Table S5.** Changes in the physical component summary score and mental component summary score of the SF-36 in patients with renal involvement. **Table S6.** Safety in patients with kidney involvement. CONSORT Checklist
