# Supplementary material for: Treatment With Avacopan in ANCA–Associated Vasculitis With Kidney Involvement
Source: Kidney Int Rep. 2025 Jun 2;10(8):2751–65. doi: 10.1016/j.ekir.2025.05.041 (PMC12348124; doi:10.1016/j.ekir.2025.05.041)
Supplement: Supplementary File (PDF) — Supplementary Methods. Table S1. Percentage of patients with active kidney manifestations on the Birmingham Vasculitis Activity Score over time. Table S2. Characteristics of patients who experienced a kidney relapse after remission. Table S3. Change in albuminuria in patients with kidney involvement. Table S4. GC use and toxicity in patients with kidney involvement. Table S5. Changes in the physical component summary score and mental component summary score of the SF-36 in patients with renal involvement. Table S6. Safety in patients with kidney involvement. CONSORT Checklist. [file mmc1.pdf]

Supplementary Material

Title

Treatment with avacopan in ANCA-associated vasculitis with kidney involvement

Authors

Duvuru Geetha, MD;[1] Frank B. Cortazar, MD;[2] Annette Bruchfeld, MD,PhD;[3,4] Andreas Kronbichler, MD; PhD[3,5,6] Alexandre Karras, MD, PhD;[7,8] Georges N. Nakhoul, MD, MEd;[9] Peter A. Merkel, MD, MPH;[10,11] Sarah Bray, PhD;[12] Alana M. Bozeman, MD;[13] David R. W. Jayne, MD[6] for the ADVOCATE Study Group[14]

Affiliations

[1]Department of Medicine, Johns Hopkins University School of Medicine, Baltimore, Maryland, USA.

[2]New York Nephrology Vasculitis and Glomerular Center, Albany, New York, USA.

[3]Department of Health, Medicine and Caring Sciences, Linköping University, Linköping, Sweden.

[4]Department of Renal Medicine, Karolinska University Hospital and CLINTEC Karolinska Institutet, Stockholm, Sweden.

[5]Department of Internal Medicine IV, Nephrology and Hypertension, Medical University Innsbruck, Innsbruck, Austria.

[6]Department of Medicine, University of Cambridge, Cambridge, UK.

[7]Université Paris Cité, Paris, France.

[8]Department of Nephrology, Hôpital Européen Georges Pompidou, APHP, Paris, France.

[9]Department of Kidney Medicine, Cleveland Clinic, Cleveland, Ohio, USA.

[10]Department of Medicine, Perelman School of Medicine, University of Pennsylvania, Philadelphia, Pennsylvania, USA

[11]Department of Biostatistics, Epidemiology, and Informatics, Perelman School of Medicine, University of Pennsylvania, Philadelphia, Pennsylvania, USA.

[12]Amgen Ltd, Cambridge, UK.

[13]Amgen Inc., Thousand Oaks, California, USA.

[14]Members of the ADVOCATE Study Group as listed in the Appendix.

Running head (44/50 characters)

Avacopan for GPA/MPA with kidney involvement

Corresponding author

Duvuru Geetha, MD; Department of Medicine, Johns Hopkins University School of Medicine, Johns Hopkins Bayview Medical Center, 301 Mason Lord Dr, Rm 2509, Baltimore, Maryland 21224, USA; dgeetha1@jhmi.edu

List of Supplementary Material

Supplementary Methods

Supplementary Table S1. Percentage of patients with active kidney manifestations on the Birmingham Vasculitis Activity Score over time

Supplementary Table S2. Characteristics of patients who experienced a kidney relapse after remission.

Supplementary Table S3. Change in albuminuria in patients with kidney involvement

Supplementary Table S4. GC use and toxicity in patients with kidney involvement

Supplementary Table S5. Changes in the Physical Component Summary Score and Mental Component Summary Score of the SF-36 in patients with renal involvement

Supplementary Table S6. Safety in patients with kidney involvement

(PDF) Supplementary information is available at KI Report's website

Supplementary Methods

As previously described,[14] patients with GPA or MPA were enrolled at 143 centers across 20 countries. Eligible patients had newly diagnosed or relapsing GPA or MPA, according to the Chapel Hill Consensus Conference definitions,[38] current or a history of a positive test result to either anti-PR3 or anti-MPO antibodies, an eGFR of at least 15 mL/min per 1.73 m^2 of body surface area, and at least 1 major or 3 minor items or at least the 2 kidney items of hematuria and proteinuria on the BVAS.[15] Randomization was performed centrally and stratified according to vasculitis disease status (newly diagnosed or relapsing), ANCA type (PR3-ANCA or MPO-ANCA), and immunosuppressive therapy (CYC [followed by AZA or MMF]) or RTX).[14]

In the study, GC treatment during the screening period had to be tapered to 20 mg or less of prednisone equivalent before the patient began the trial; this open-label GC treatment was further tapered to discontinuation by the end of week 4 of the trial.[14] Patients in either treatment group who had a worsening of disease that involved a major item in the BVAS could be administered rescue therapy consisting of i.v. GC (typically 0.5–1 g of methylprednisolone per day for 3 days), oral GCs, or both, tapered according to the patient's condition.

Avacopan (30 mg two times per day) or matching placebo was administered for 52 weeks to patients randomized (1:1) using an interactive web-response system, with the use of a minimization algorithm, to maintain balance between the treatment groups.[14] Prednisone or a matched placebo was administered on a tapering schedule over 20 weeks (60 mg per day tapered to discontinuation by week 21). RTX was administered i.v. at a target dose of 375 mg/m^2 of body surface area per week in the first 4 weeks; patients on induction therapy did not receive any RTX therapy to maintain remission. CYC was administered via i.v. at a target dose of 15 mg/kg of body weight up to 1.2 g on day 1 and at weeks 2, 4, 7, 10, and 13 or orally at a dose of 2 mg/kg up to 200 mg/day for 14 weeks. From week 15 onward, CYC was followed by oral AZA at a target dose of 2 mg/kg per day or, if AZA was not tolerated, MMF at a target dose of 2 g/day. The study included a 52-week treatment period and 8-week follow up period.

Efficacy endpoints were evaluated over the 52-week treatment period. Safety outcomes were evaluated over the full 60-week study duration.

Supplementary Table S1. Percentage of patients with active kidney manifestations of vasculitis over time

Treatment	Kidney Manifestation, *n* (%)	Study Week						
		Baseline	4	10	16	26	39	52
Prednisone taper (*n* = 134)	*N*1	134	132	131	128	127	126	125
	Overall	134 (100.0)[a]	19 (14.4)	10 (7.6)	8 (6.3)	6 (4.7)	1 (0.8)	4 (3.2)
	RBC casts and/or glomerulonephritis	61 (45.5)	6 (4.5)	3 (2.3)	3 (2.3)	2 (1.6)	0 (0.0)	1 (0.8)
	Hypertension	23 (17.2)	1 (0.8)	1 (0.8)	1 (0.8)	2 (1.6)	0 (0.0)	0 (0.0)
	Proteinuria > 1+ or > 0.2 g/g creatinine	106 (79.1)	15 (11.4)	4 (3.1)	4 (3.1)	4 (3.1)	1 (0.8)	4 (3.2)
	Hematuria ≥ 10 RBCs/hpf	72 (53.7)	12 (9.1)	7 (5.3)	6 (4.7)	1 (0.8)	1 (0.8)	3 (2.4)
	Serum creatinine ≥ 125 µmol/L	62 (46.3)	0 (0.0)	0 (0.0)	0 (0.0)	0 (0.0)	0 (0.0)	0 (0.0)
	Rise in serum creatinine > 30% or fall in creatinine clearance > 25%	19 (14.2)	3 (2.3)	2 (1.5)	2 (1.6)	3 (2.4)	1 (0.8)	1 (0.8)
	Other	0 (0.0)	0 (0.0)	0 (0.0)	0 (0.0)	0 (0.0)	0 (0.0)	0 (0.0)
Avacopan (*n* = 134)	*N*1	134	133	128	128	127	124	123
	Overall	134 (100.0)[a]	27 (20.3)	9 (7.0)	7 (5.5)	3 (2.4)	2 (1.6)	1 (0.8)
	RBC casts and/or glomerulonephritis	63 (47.0)	6 (4.5)	3 (2.3)	1 (0.8)	1 (0.8)	1 (0.8)	1 (0.8)
	Hypertension	20 (14.9)	4 (3.0)	1 (0.8)	2 (1.6)	1 (0.8)	2 (1.6)	0 (0.0)
	Proteinuria > 1+ or > 0.2 g/g creatinine	109 (81.3)	19 (14.3)	8 (6.3)	6 (4.7)	2 (1.6)	1 (0.8)	1 (0.8)
	Hematuria ≥ 10 RBCs/hpf	79 (59.0)	18 (13.5)	6 (4.7)	4 (3.1)	1 (0.8)	0 (0.0)	0 (0.0)
	Serum creatinine ≥ 125 µmol/L	59 (44.0)	0 (0.0)	0 (0.0)	0 (0.0)	0 (0.0)	0 (0.0)	0 (0.0)
	Rise in serum creatinine > 30% or fall in creatinine clearance > 25%	27 (20.1)	1 (0.8)	3 (2.3)	2 (1.6)	0 (0.0)	1 (0.8)	0 (0.0)
	Other	1 (0.7)	0 (0.0)	0 (0.0)	0 (0.0)	0 (0.0)	0 (0.0)	0 (0.0)

[a]As assessed by investigators.
N1 = number of patients evaluated at that time point.
hpf, high powered field; RBC, red blood cell.

Supplementary Table S2. Characteristics of patients who experienced a kidney relapse after remission[a]

	Hematuria (RBCs/hpf)	UACR (mg/g)	Kidney Relapse After Remission[a] (per the BVAS)							
			Hematuria		Proteinuria[b]		Rise in creatinine[c]		Hypertension	
**Treatment**	**Week 26**	**Week 26**	**Week 39**	**Week 52**	**Week 39**	**Week 52**	**Week 39**	**Week 52**	**Week 39**	**Week 52**
Prednisone taper										
Patient 1	16–29	161	–	–	–	Yes	–	–	–	–
Patient 2	16–29	426	–	Yes	–	Yes	–	Yes	–	–
Patient 3	> 75	727	Yes	–	Yes	–	Yes	–	–	–
Patient 4	16–29	1731	–	Yes	–	Yes	–	–	–	–
Patient 5	1–2	159	–	Yes	–	Yes	–	–	–	–
Avacopan										
Patient 6	> 75	362	–	–	Yes	–	Yes	–	Yes	–
Patient 7	–	9	–	–	–	–	–	–	Yes	–
Patient 8	3–5	1108	–	–	–	Yes	–	–	–	–

[a]Kidney relapse after remission is defined as recurrence of a kidney manifestation of vasculitis (per the BVAS) at week 39 and/or 52 after the primary endpoint of remission was achieved at week 26.
[b]Proteinuria > 1+ or > 0.2 g/g creatinine.
[c]Rise serum creatinine > 30% or fall in creatinine clearance > 25%.
BVAS, Birmingham Vasculitis Activity Score; hpf, high powered field; RBC, red blood cell; UACR, urinary albumin-to-creatinine ratio.

Supplementary Table S3. Change in albuminuria in patients with kidney involvement

	**Prednisone Taper** ***n* = 134**	**Avacopan** ***n* = 134**	**Difference (95% CI)**
Baseline UACR > 300 mg/g			
Baseline, geometric mean (range), mg/g [*N*1]	820 (306–5367) [74]	903 (323–6461) [81]	
Percentage change from baseline (%), LS mean ± SEM [*N*1]			
Week 2	2 ± 11 [74]	–28 ± 11 [78]	–29 (–48, –5)
eGFR ≥ 90[a]	2 ± 106 [3]	–66 ± 75 [5]	–67 (–95, 137)
eGFR 60–89[a]	44 ± 72 [4]	–37 ± 50 [7]	–57 (–90, 82)
eGFR 45–59	–13 ± 36 [11]	–39 ± 40 [9]	–30 (–72, 76)
eGFR 30–44	–11 ± 23 [17]	–32 ± 19 [23]	–24 (–56, 30)
eGFR 15–29	10 ± 16 [38]	–18 ± 17 [33]	–26 (–51, 13)
Week 4	–5 ± 12 [71]	–44 ± 11 [78]	–41 (–56, –20)
eGFR ≥ 90[a]	–10 ± 106 [3]	–83 ± 75 [5]	–81 (–97, 35)
eGFR 60–89[a]	6 ± 72 [4]	–58 ± 50 [7]	–61 (–91, 65)
eGFR 45–59	–31 ± 36 [11]	–39 ± 40 [9]	–11 (–65, 124)
eGFR 30–44	–7 ± 23 [16]	–35 ± 19 [23]	–30 (–60, 20)
eGFR 15–29	5 ± 16 [36]	–41 ± 17 [33]	–44 (–63, –15)
Week 13	–48 ± 12 [70]	–55 ± 11 [76]	–14 (–37, 15)
eGFR ≥ 90[a]	–76 ± 106 [3]	–81 ± 75 [5]	–18 (–88, 486)
eGFR 60–89[a]	–59 ± 72 [4]	–62 ± 50 [7]	–8 (–78, 287)
eGFR 45–59	–66 ± 36 [11]	–51 ± 40 [9]	43 (–43, 263)
eGFR 30–44	–56 ± 23 [16]	–55 ± 19 [23]	2 (–41, 76)
eGFR 15–29	–27 ± 16 [35]	–53 ± 17 [31]	–36 (–58, –1)
Week 26	–68 ± 12 [68]	–67 ± 11 [72]	5 (–23, 42)
eGFR ≥ 90[a]	–79 ± 106 [3]	–86 ± 75 [5]	–34 (–91, 372)
eGFR 60–89[a]	–67 ± 72 [4]	–71 ± 50 [7]	–12 (–79, 269)
eGFR 45–59	–64 ± 36 [11]	–62 ± 41 [8]	11 (–57, 182)
eGFR 30–44	–78 ± 23 [17]	–58 ± 19 [23]	92 (12, 230)
eGFR 15–29	–61 ± 16 [32]	–71 ± 17 [29]	–26 (–52, 15)
Week 52	–75 ± 12 [68]	–75 ± 12 [70]	1 (–26, 38)
eGFR ≥ 90[a]	–86 ± 106 [3]	–89 ± 75 [5]	–25 (–89, 437)
eGFR 60–89[a]	–67 ± 76 [3]	–75 ± 51 [6]	–24 (–83, 231)
eGFR 45–59	–73 ± 36 [11]	–65 ± 42 [8]	30 (–50, 234)
eGFR 30–44	–79 ± 23 [17]	–72 ± 20 [22]	36 (–21, 135)
eGFR 15–29	–72 ± 17 [33]	–78 ± 18 [29]	–22 (–50, 21)
Baseline UACR > 300 mg/g and baseline adjudicated BVAS item rise in serum creatinine > 30% or fall in creatinine clearance > 25%			
Baseline, geometric mean (range), mg/g [*N*1]	951 (456–3197) [11]	926 (356–2280) [13]	
Percentage change from baseline (%), LS mean ± SEM [*N*1]			
Week 2	10 ± 29 [11]	4 ± 26 [13]	–6 (–53, 87)
Week 4	14 ± 29 [11]	–21 ± 26 [13]	–30 (–65, 39)
Week 13	–24 ± 29 [11]	–39 ± 26 [13]	–21 (–60, 58)
Week 26	–55 ± 29 [11]	–53 ± 27 [12]	6 (–47, 112)
Week 52	–75 ± 29 [11]	–68 ± 27 [12]	29 (–36, 160)

N1 = number of patients evaluated.
[a]Imprecise estimations due to limited patient number.
eGFR, estimated glomerular filtration rate; hpf, high powered field; LS, least squares; SEM, standard error of mean; UACR; urinary albumin-to-creatinine ratio.

Supplementary Table S4. Glucocorticoid use and glucocorticoid toxicity in patients with kidney involvement

	Prednisone Taper<br>*n* = 134	Avacopan<br>*n* = 134	Difference<br>(95% CI)[a]
**Glucocorticoid Use**			
Total all-source glucocorticoid dose,[b] mg prednisone dose equivalent[c]			
Weeks 0 to 4			
n/*N*1 (%)	134/134 (100.0)	108/134 (80.6)	
Mean ± SD	1612 ± 547	598 ± 1221	
Median (min, max)	1518 (760, 5355)	396 (0, 12677)	
Weeks 0 to 26			
n/*N*1 (%)	134/134 (100.0)	113/134 (84.3)	
Mean ± SD	3319 ± 1363	1276 ± 2291	
Median (min, max)	2950 (760, 11815)	500 (0, 19492)	
Weeks 27 to 52			
n/*N*1 (%)	51/128 (39.8)	37/126 (29.4)	
Mean ± SD	505 ± 1006	333 ± 781	
Median (min, max)	0 (0, 6333)	0 (0, 4565)	
Weeks 0 to 52			
n/*N*1 (%)	134/134 (100.0)	115/134 (85.8)	
Mean ± SD	3801 ± 1934	1589 ± 2678	
Median (min, max)	3028 (760, 13383)	576 (0, 21680)	
Nonstudy supplied i.v. and oral glucocorticoid dose, mg prednisone dose equivalent[c]			
Screening (weeks –2 to 0)			
n/*N*1 (%)	111/134 (82.8)	99/134 (73.9)	
Mean ± SD	1038 ± 1195	996 ± 1191	
Median (min, max)	535 (0, 4465)	460 (0, 5805)	
Weeks 0 to 4			
n/*N*1 (%)	112/134 (83.6)	84/134 (80.6	
Mean ± SD	465 ± 539	598 ± 1221	
Median (min, max)	400 (0, 4225)	396 (0, 12677)	
Weeks 0 to 26			
n/*N*1 (%)	119/134 (88.8)	113/134 (84.3)	
Mean ± SD	934 ± 1258	1276 ± 2291	
Median (min, max)	500 (0, 6375)	500 (0, 19492)	
Weeks 27 to 52			
n/*N*1 (%)	51/128 (39.8)	37/126 (29.4)	
Mean ± SD	505 ± 1006	333 ± 781	
Median (min, max)	0 (0, 6333)	0 (0, 4565)	
Weeks 0 to 52			
n/*N*1 (%)	119/134 (88.8)	115/134 (85.8)	
Mean ± SD	1416 ± 11862	1589 ± 2678	
Median (min, max)	625 (0, 9950)	575 (0, 21680)	
Nonstudy supplied i.v. glucocorticoid dose, mg prednisone dose equivalent[c]			
Screening (weeks –2 to 0)			
n/*N*1 (%)	67/134 (50.0)	56/134 (41.8)	
Mean ± SD	849 ± 1150	810 ± 1179	
Median (min, max)	63 (0, 3750)	0 (0, 5625)	
Weeks 0 to 4			
n/*N*1 (%)	86/134 (64,1)	84/134 (62.7)	
Mean ± SD	330 ± 462	384 ± 601	
Median (min, max)	250 (0, 3750)	215 (0, 4250)	

	Prednisone Taper *n* = 134	Avacopan *n* = 134	Difference (95% CI)[a]
Weeks 0 to 26			
n/*N*1 (%)	94/134 (70.1)	91/134 (67.9)	
Mean ± SD	472 ± 797	599 ± 1039	
Median (min, max)	396 (0, 5725)	375 (0, 6250)	
Weeks 27 to 52			
n/*N*1 (%)	15/128 (11.7)	12/125 (9.6)	
Mean ± SD	74 ± 371	49 ± 233	
Median (min, max)	0 (0, 3125)	0 (0, 1875)	
Weeks 0 to 52			
n/*N*1 (%)	97/134 (72.4)	92/134 (68.7)	
Mean ± SD	543 ± 867	645 ± 1083	
Median (min, max)	400 (0, 5725)	384 (0, 6250)	
**Glucocorticoid Toxicity Index**			
GTI-Cumulative Worsening Score,[d] LS mean (95% CI)[a] [*N*1]			
Week 13	37.7 (29.9, 45.5) [131]	24.1 (16.5, 31.8) [132]	–13.5 (–23.4, –3.7)
Week 26	58.5 (50.6, 66.4) [126]	38.9 (31.2, 46.6) [125]	–19.6 (–29.6, –9.6)
GTI-Aggregate Improvement Score,[e] LS mean (95% CI)[a] [*N*1]			
Week 13	24.3 (16.4, 32.1) [131]	8.5 (0.8, 16.2) [132]	–15.7 (–25.7, –5.8)
Week 26	24.3 (16.4, 32.2) [126]	11.2 (3.4, 19.0) [125]	–13.1 (–23.2, –3.0)

N1 = number of patients evaluated.
[a]Week 13 and Week 26 LS means and 95% CIs were from MMRM models that incorporate treatment group, visit, treatment-by-visit interaction and stratification factors (newly diagnosed or relapsed ANCA-associated vasculitis, anti-PR3 or anti-MPO ANCA, and i.v. rituximab, or cyclophosphamide (i.v. or oral) as covariates.
[b]Total is for study supplied and non-study supplied glucocorticoids and includes both i.v. and oral glucocorticoids.
[c]All doses were converted to prednisone equivalent (mg) and are calculated as total dose during a specified period. The *n* (%) data are the number of patients who used any GCs during the period and the mean and median (range) data are for all patients in the period.
[d]Score ranges from 0 to 410, with higher scores indicating greater severity of toxic effects.
[e]Score ranges from –317 to 410, with higher scores indicating greater severity of toxic effects.
ANCA; antineutrophil cytoplasmic antibody; CI, confidence interval; GTI, Glucocorticoid Toxicity Index; i.v., intravenous; LS, least squares; max, maximum; min, minimum; MMRM, mixed-effects models for repeated measures; MPO, myeloperoxidase; PR3, proteinase 3; SD, standard deviation.

Supplementary Table S5. Changes in the Physical Component Summary Score and the Mental Component Summary Score of the 36-Item Short Form Health Survey in patients with kidney involvement

**Item**	**Prednisone Taper** ***n* = 134**	**Avacopan** ***n* = 134**	**Difference (95% CI)**
Physical Component Summary Score			
Baseline, mean (SD) [N1]	40.4 (10.3) [130]	39.5 (10.7) [133]	
Change from baseline, LS mean (95% CI)			
Week 26	1.9 (0.3–3.6) [120]	4.8 (3.2–6.4) [124]	2.9 (0.8–5.0)
Week 52	3.1 (1.5–4.8) [117]	4.9 (3.3–6.5) [120]	1.8 (–0.3 to 4.0)
Mental Component Summary Score			
Baseline, mean (SD) [N1]	42.0 (13.4) [130]	44.5 (12.8) [134]	
Change from baseline, LS mean (95% CI)			
Week 26	3.5 (1.6–5.4) [120]	5.2 (3.3–7.0) [125]	1.7 (–0.8 to 4.1)
Week 52	5.1 (3.2–7.0) [117]	6.6 (4.8–8.5) [120]	1.5 (–1.0 to 4.0)

N1 = number of patients evaluated.
CI, confidence interval; LS, least squares; SF-36, 36-item short form health survey; SD, standard deviation.

Supplementary Table S6. Safety in patients with kidney involvement

Item	Prednisone Taper<br>*n* = 134	Avacopan<br>*n* = 134
Overall AEs		
Patients, *n* (%)	133 (99.3)	132 (98.5)
Events	1777	1465
SAEs		
Patients, *n* (%)	65 (48.5)	61 (45.5)
Events	148	104
Discontinuation of blinded medication		
Patients, *n* (%)	20 (14.9)	24 (17.9)
Infections		
Patients, *n* (%)	103 (76.9)	91 (67.9)
Events	241	200
Serious infections		
Patients, *n* (%)	24 (17.9)	20 (14.9)
Events	30	23
Serious AEs of abnormality on liver-function testing[a]		
Patients, *n* (%)	5 (3.7)	8 (6.0)
Required dialysis, *n* (%)	4 (3.0)[b]	4 (3.0)[b]
Death, *n* (%)	3 (2.2)	2 (1.5)

[a]Defined as serious treatment-emergent AEs with preferred terms of hepatic enzyme increased, alanine aminotransferase increased, aspartate aminotransferase increased, blood bilirubin increased, liver function test increased, transaminases increased, liver function test abnormal, hepatic function abnormal, drug-induced liver injury, hepatitis cholestatic, and hepatocellular injury.

[b]Four patients in the prednisone taper group received dialysis (permanent in 2 patients, temporary in 1 patient, and additional information was not available for 1 patient whose eGFR was 7 mL/min/1.73 m^2 at their study end [day 184]). Three patients in the avacopan group received dialysis (permanent in 2 patients and a single session in 1 patient) and dialysis was planned in 1 more patient (expected to be permanent).

AE, adverse event; SAE, serious adverse event.
